# Towards an Ultrasonic Guided Wave Procedure for Health Monitoring of Composite Vessels: Application to Hydrogen-Powered Aircraft

**DOI:** 10.3390/ma10091097

**Published:** 2017-09-19

**Authors:** Slah Yaacoubi, Peter McKeon, Weina Ke, Nico F. Declercq, Fethi Dahmene

**Affiliations:** 1 Institut de Soudure, 4 Bvd Henri Becquerel, Espace Cormontaigne, 57970 Yutz, France; w.ke@isgroupe.com (W.K.); f.dahmene@isgroupe.com (F.D.); 2 Unité Mixte Internationale Georgia Tech—CNRS 2958, George W. Woodruff School of Mechanical Engineering, Metz Technopole, 2 Rue Marconi, 57070 Metz, France; peter.mckeon@georgiatech-metz.com (P.M.); nico.declercq@me.gatech.edu (N.F.D.)

**Keywords:** composite over-wrapped pressure vessel, hydrogen safety, structural health monitoring, ultrasonic guided waves technique, testing procedure, finite element method

## Abstract

This paper presents an overview and description of the approach to be used to investigate the behavior and the defect sensitivity of various ultrasonic guided wave (UGW) modes propagating specifically in composite cylindrical vessels in the framework of the safety of hydrogen energy transportation such as hydrogen-powered aircrafts. These structures which consist of thick and multi-layer composites are envisioned for housing hydrogen gas at high pressures. Due to safety concerns associated with a weakened structure, structural health monitoring techniques are needed. A procedure for optimizing damage detection in these structural types is presented. It is shown that a finite element method can help identify useful experimental parameters including frequency range, excitation type, and receiver placement.

## 1. Introduction

Hydrogen-powered aircrafts are increasingly developing, and some prototypes are already deployed [1,2]. Waste water and urine available on-board can be used to generate hydrogen, which is an ecological and economic solution [3]. Hydrogen has shown promise as an alternative fuel source for transportation vehicles and accordingly, high-pressure hydrogen storage vessels have been developed to be light-weight so as to be easily transportable [4,5,6,7].

A popular design of the reservoirs involves a thin polyamide liner that prevents the diffusion of hydrogen, and a thicker carbon fiber reinforced plastic (CFRP) over-wrap which gives the vessel the strength needed to withstand the high internal pressure [8]. This kind of structure, commonly called Composite Over-wrapped Pressure Vessel (COPV) [1,2,9], is chosen for several characteristics, e.g., their high strength-to-weight ratio. The structural integrity and strength of such COPVs are due mainly to the integrity of fibers [10,11,12]. Therefore, a rupture of a group of fibers (as is the case for a cut or gouge) would be critical to the overall integrity of the structure, especially when it arises on its surface. The CFRP composite has almost no plastic ductility, thus low fracture toughness, which makes the structure highly sensitive to minor surface defects or damage [13]. This highlights the need for nondestructive testing and monitoring (NDT & M) techniques to continuously assess the high-pressure COPVs’ structural integrity, especially since they are envisioned for use in public transportation systems [4,14]. Early detection of failure modes is a key method of alleviating safety concerns [15,16].

Ultrasonic Guided Waves (UGW) technique is one among several NDT & M techniques, and has many properties ideal for complicated endeavors [17]. Intrinsically, UGWs travel along waveguides such as plates, pipes, rods, etc., covering larger distances than traditional tools such as C-scans which inspect these structures point by point [18]. Furthermore, unlike C-scans, UGW techniques have the potential of being permanently attached to the structure in question, which makes them ideal for monitoring. Recently, the application range of the UGW technique has been expanded to more complex materials and geometries, with considerable effort being directed at implementing these systems for the purpose of damage detection in anisotropic composites [19,20].

Controlled experimental setups have the advantage of realistically simulating in-situ field use, but if numerical models have been justly verified, they can be effectively used as an optimization tool. The use of Finite Element Modeling (FEM) is highly beneficial for the optimization of procedures whose purpose is identifying different damage types. In the past, FEM results have been used to help select the most efficient methods of excitation and reception so as to identify which UGW modes are sensitive to various damage types and orientations as demonstrated by the seminal work of Alleyne and Cawley [21]. Use of FEM as an optimization tool can also save time and money, since many parameters can be easily adjusted within the model, which would prove time-consuming and expensive in actual experimental setups. Once all parameters (or most of them) are determined and verified experimentally, establishing an industrial procedure will be a straightforward item.

The goal of this work is to give a numerical overview-investigation of the propagation of UGW in a cylindrical COPV, which is a complex structure with regard to propagation phenomena (dispersive and multimodal behaviors, multipath, multi-reflection, attenuation, etc.). The underlying purpose is to provide the reader with insight into what phenomena take place, how they influence the sensitivity to damage detection and how this may, in general, help the reader to make decisions concerning the specific UGW technique/mode to use in order to optimize the detectability. In other words, the investigations will help, in general, understand the waves’ propagation in such a COPV, and consequently can serve to optimize experimental parameters so as to carry out a reliable testing/monitoring procedure. In order to gain a deeper understanding of this work, the next section is devoted to a concise background of UGW. In the same section, a description of the scope of the current work is detailed. Section 3 presents an in-depth discussion of the problem at hand, and describes the models used to predict modal conversion and optimize some of the most essential parameters. Corresponding results and discussions are pointed out in the same section. Finally, Section 4 is concerned with the main concluding remarks of the presented modeling work and provides some outlooks.

## 2. Background and Scope of the Work

To ease understanding to the non-specialist reader, terminologies, needed to better understand the current topic, and to assess the real need of this study, are introduced and described in this section. Most of these key terminologies are italicized.

UGWs are waves which propagate through the wall-thickness of the structure (COPV in the present study) to be tested or monitored, at frequencies higher than 20 kHz. At least one emitter and one receiver are needed to send and receive signals. These signals are captured after propagation, post-processed, and then analyzed to examine the integrity of the structure. It is possible to use transducers (i.e., emitter and receiver) in two configurations: *pulse-echo* and *pitch-catch* [22]. In either case, the presence of damage sites may be detected, classified, and localized by analyzing the received signal(s). To do so, many parameters need to be optimized to carry out this work effectively. *Frequency range*, *excitation type* and *receiver placement* are some of the most important parameters to be ascertained.

**Figure 1:**
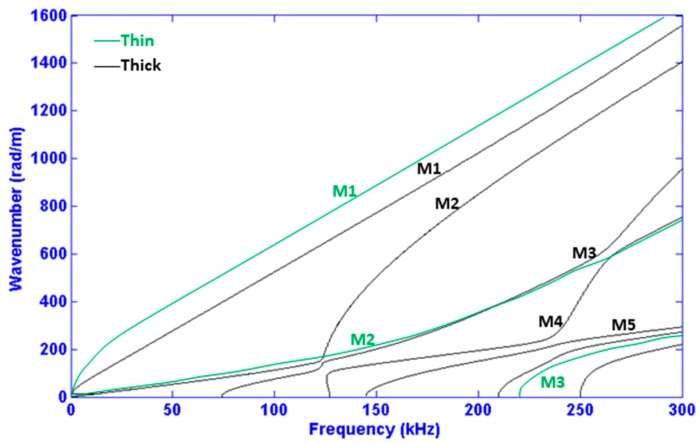
Dispersion curves in two cases of COPV thicknesses: thin (black) and thick multi-layer (green) with a thickness equal two times that of the thinner one (5 mm). M*_i_* is the *i*th mode where *i* represents its order.

For the purpose of introducing and understanding some other key concepts in the field of NDT & M via UGW, we may consider Figure 1. This figure compares the *dispersion curves* of a thin COPV with those of a thick multi-layer COPV. The most obvious discrepancy between the two results is that the thick multi-layer COPV has more *modes* and those present have more complicated shapes. In sharp contrast, a *non-dispersive* signal would behave linearly in the frequency-wavenumber plane. In general, one could re-scale a problem by simultaneously reducing both the wall-thickness and the applied acoustic wavelength. The reason why such re-scaling is not applied here is that defects normally cannot be scaled. So, while we consider thick layered structures, we must maintain the relatively short wavelengths (or high frequencies) to preserve the detectability of defects. For this reason, the appearance of more modes for thick plates is a practical problem that cannot be overcome. Therefore, frequency range, enforced by the need for precision to detect defects of given sizes, can impact how many modes can possibly propagate in a given COPV. In addition, making an *operational frequency* choice does not just simply limit the number of propagating modes; an intelligent choice can also simplify a received signal and must clearly be considered whenever possible. Consequently, choosing a frequency span that corresponds to relatively *non-dispersive* UGW modes can help limit temporal *signal spreading* due to dispersion [23]. The importance of the frequency range choice also manifests itself in terms of *mode shapes* since in general UGW mode shapes are a function of frequency. The stress and displacement across the thickness of the structure (i.e., COPV in the current case) inform which modes are capable of being excited or detected from the top surface, and with which kind of damage they will most strongly interact.

A structural health monitoring system is by definition a live-in system, and therefore UGW must be excited and detected by some permanently attached transducer system. Accordingly, this study will limit itself to contact transducers, flush against the outer-surface of the COPV. This configuration lacks some of the advantages when compared to non-contact transducers, most notably phase matching by an obliquely incident bounded beam [16,24]. However, the benefit of having a live-in system is essential: structural integrity can be continuously monitored. Therefore, the evolution of damage severity can be monitored in real-time, and consequently COPVs can be replaced before defects grow to critical sizes.

Some other restrictions are self-imposed to help limit the scope of the present work. Only out-of plane displacement on the top surface of the structure will be monitored as a means of reception, since out-of-plane motion on the surface of the plate is not only accessible, but a common parameter monitored by already existing NDT & M methods [25]. Damage detection will be accomplished via the appearance (or change in amplitude) of modes in the transmission and reflection field after the incident wave interacts with the damage site. Propagating modes can be distinguished by the receiving method, and can be accomplished via a variety of means, for example, through intelligent angling of air-coupled transducers according to Snell’s law [26], applying a short-time Fourier transform to a single waveform [27], or a two-dimensional Fourier transform to a series of evenly spaced waveforms [28], which coincidently is how modes are separated in this work.

To further limit the scope of the paper, the damage types will be limited to surface-originating cracks which share a similar geometry to cuts or gauges [29]. Note that, in general, similar procedures to the one developed in this work can be applied to other damage types.

Besides, FEM can be performed in either the time domain or frequency domain [30], and in two dimensions (2D) or three dimensions (3D). Time and frequency domain methods of calculation are different and complementary. The former is time-consuming but it allows us to mimic the propagation (i.e., visioning waves’ displacement) while the latter permits getting a relatively quick result for a single frequency. In the present work, regarding its objectives, the two methods are considered.

## 3. Problem Statement

**Figure 2:**
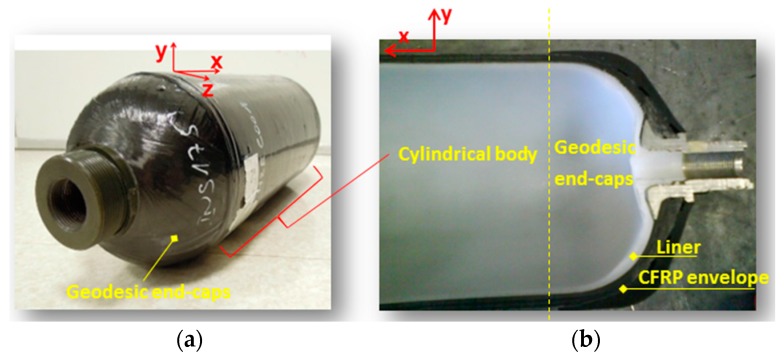
Example of a multi-layer COPV intended for housing hydrogen gas at high pressures: overview (**a**) and partial sectional view (**b**).

Consider a structure consisting of a cylindrical body with two spherical end-caps, comprising of two layers: a thin ‘macroscopically isotropic’ liner and a thicker ‘macroscopically anisotropic’ layer, as shown in Figure 2. Although, the spherical end caps may also undergo damage, this present work will focus mainly on the cylindrical central portion of the reservoir that makes up the majority of its surface area, as shown in the same figure. In addition, the circumferential stress in the cylindrical part is roughly two times the stress elsewhere [2] and so, a given damage in this part should be more severe than in end-caps.

In addition, COPVs exist in industry in various volumes (i.e., different dimensions). If the radius-to-thickness ratio is large enough, the problem can be simplified from a cylindrical configuration to approximate that of a plate [31,32]. In the current case, the diameter is 100 mm and the thickness is 10 mm. The model can be simplified further from 3D to 2D by taking advantage of material symmetry. This simplification is possible if the UGW being modeled is a plane wave traveling in a direction where the energy propagation vector is perpendicular to the surface of the traveling plane wave front. This occurs due to material symmetry in orthotropic materials when the propagation direction is aligned with any of the principal directions of the medium.

In the 2D model, computations are chosen to be carried out in the frequency domain, thus greatly reducing computational time. Therefore, the Helmholtz equation is solved in lieu of the wave equation. This equation is given by:

(1) $$C_{ijkl}\frac{\partial^{2}u_{l}}{\partial x_{j}\partial x_{k}}-\rho \omega^{2}u_{i}=0\;i,j,k,l=1,2,3$$

where *C_ijkl_* is the material’s stiffness matrix and can be composed of complex moduli; *i*, *j*, *k* and *l* are indices referring to one of the three Cartesian coordinates; *u_q_* is the displacement in the direction given by its subscript *q*; and *ρ* and ω are density and angular frequency, respectively.

**Figure 3:**
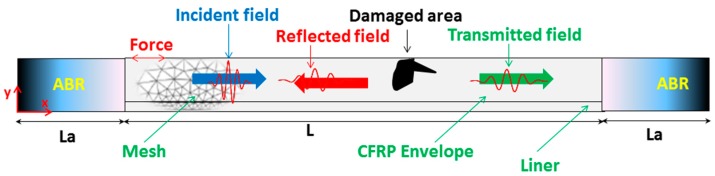
Schematic of the Finite Element simulation model (ABR: Absorbing Region of a length La).

A small portion of the boundary is subject to forces that act as the excitation source. The propagation of UGW is along the *x*-direction and the surface of the plate structure is perpendicular to the *y*-direction, i.e., in the *z*-direction. For convenience, the origin of the Cartesian reference frame is placed at the interface between the two layers. Here, the plate is considered infinite in the *z*-direction. Concerning the *x*-direction, the propagation domain (PD) is finite, and of length L, as shown in Figure 3.

To eliminate wave reflections by the extremities of the PD, Absorbing Boundary Regions (ABR) or some similar boundary setting to dampen unwanted reflections must be added. In a previous work [33], a comparative study concerning the ABR models that have been developed in the past was carried out. The optimal solution is to use ABR’s that increase their dampening capabilities cubically as per the work of Hosten and Castaings [34], and improved later by Ke et al. [35].

Moreover, end-caps (i.e., geodesic form) complicate the shape of the signals to be collected and so, impact the wave propagation features that are needed to determine the pass-fail criteria of COPVs. This is because UGW is sensitive, in nature, to geometrical characteristics, which can undergo multiple propagation paths [36,37]. To complement the frequency modelling done in 2D, simulations in 3D will be carried out in the time domain (see [27] for further details). Although mechanical properties influence waves’ propagation, the 3D models are simplified by using isotropic stainless steel as material to get a first glimpse of what may occur. This takes into consideration only geometry as the main influence factor in our 3D studies. The model in this study uses a given geometry with a diameter of 100 mm, 600 mm cylinder body length and thickness of 3 mm. Simulations are performed for two different kinds of excitation shape. In both cases, the excitation is localized on the top side and near the extremity of the cylindrical part of the COPV. Further details are given in Section 4.5.

The actual material properties are subject to a non-disclosure restriction. However, in order to understand the results in this paper, we can mention that the lay-up (inside to outside) consists of a polyamide liner, a carbon-fiber reinforced polymer overwrap and a glass fiber reinforced polymer shell. The polyamide layer is very thin and can be represented by typical nylon-type material properties. The carbon fiber reinforced polymer overwrap shows orthotropic stiffness behavior with principle symmetry directions along the axial (*x*), the radial (*y*) and the tangential (*z*) cylindrical directions. The glass fiber reinforced polymer shell acts as a protective shell which is also orthotropic and principle directions equal to those of the carbon fiber reinforced polymer. The relative wall thicknesses are mentioned, as needed, elsewhere in the paper. The presented numerical simulations have been preceded by convergence tests to ensure that the mesh size was sufficiently small to allow correct simulations; more particularly, we ensured that the difference between the presented monitored quantities between two successive mesh sizes was less than 2%.

## 4. Results and Discussions

### 4.1. Mode Selection

**Figure 4:**
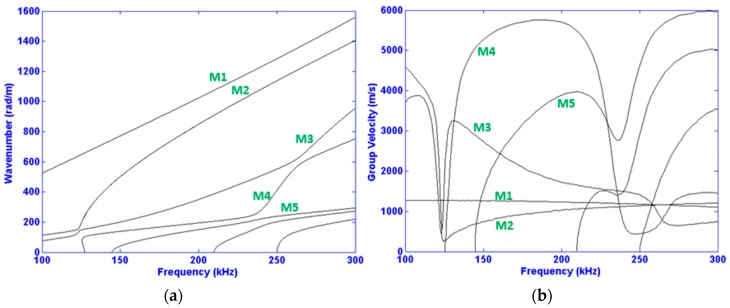
Wavenumber (**a**) and group velocity (**b**) dispersion curves determined along the direction of propagation (*x*) for the two-layer structure shown in Figure 3. M*_i_* denotes modes *i* with *i* = 1, 2, 3, 4 and 5.

The relevant dispersive relations of wavenumber and group velocity are displayed in Figure 4. They are determined via the commercial software Disperse^®^ [38] for the frequency range of interest, i.e., 100–300 kHz. This frequency range is practical in that it helps limit the number of total propagating modes. The higher the frequency range, the more modes appear, indefinitely, as testified by the appearance of higher order modes at higher frequencies in Figure 4. Moreover, the lower order modes (Modes 1 and 2) range an order of magnitude of wavelengths from 4 to 30 mm, as can be deduced from Figure 4 left. In other words, this range is favorable in order to have a relatively simple signal and satisfactory sensitivity. Sensitivity is often found to be directly correlated to wavelength size [20].

**Figure 5:**
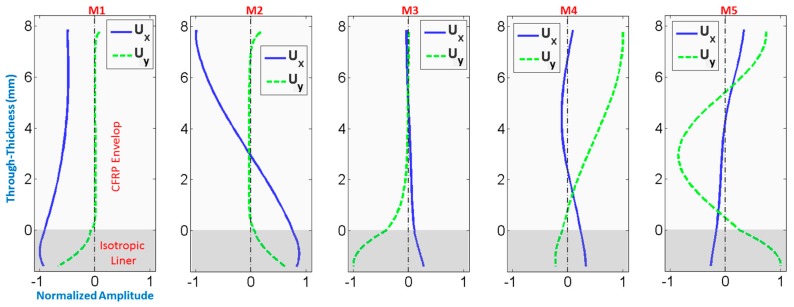
Mode shapes calculated for a specific case of COPV (2 layers having thicknesses 7.85 mm and 1.39 mm respectively) at around 180 kHz for Modes 1 through 5, from left to right.

As stated in Section 2, mode shapes change with frequency. To illustrate, mode shapes at around 180 kHz (medium value of the range of interest) are depicted in Figure 5 for the first 5 modes. The optimization of mode selection is strongly related to the optimization of the operational frequency, which will be discussed hereafter. Instead of looking at the evolution of each propagating mode as frequency increases from 100 to 300 kHz, it is more efficient to model the interaction of the mode shape with a defect site, as a function of frequency. Evidently, modeling defects is in itself a complex and interesting problem. For the sake of brevity, the present work will consider a surface-originating crack modeled as a notch, which is the most severe kind of defect as explained in the introduction section.

### 4.2. Transducer Vibration Mode

**Figure 6:**
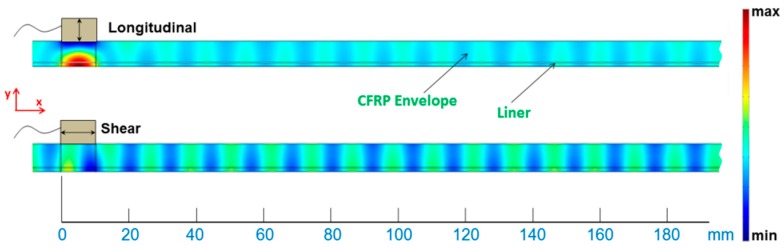
Typical FEM simulation results for longitudinal (**top**) and shear (**bottom**) mode transducer types shown on the same scale, for excitation frequency of 200 kHz.

One of the defining characteristics of piezoelectric transducers is their vibrational mode. Commercial piezoelectric transducers are available in both shear (parallel to the surface of the plate) and longitudinal (perpendicular to the surface of the plate) configurations [39,40]. In the FEM frequency domain simulations, these two excitation types can be modeled as a boundary force acting over the characteristic dimension of the transducer. The boundary force excitation is located between 0 and 10 mm on the top surface, as schematized in Figure 6. The restriction to the upper surface is necessary for practical reasons (surface of the COPV). This figure shows the results of the FEM simulation in terms of out-of-plane displacement as a way of qualitatively comparing the two excitation types at 200 kHz. These images show a zoom on one portion of the model to more easily show the excited mode shapes.

A standing wave established between the top and bottom surface within the confines of the bounded beam profile of the contact transducers was expected. The excitation types mimic contact transducers (both shear and longitudinal) fixed flush to the surface of the waveguide, and therefore cannot take advantage of the phase matching effect that angular wedges or comb transducers may exploit. Accordingly, any UGW’s that are propagated in the x-direction are a result of waves escaping the bounded beam profile of the transducer, due mainly to material stiffness. Although both methods succeed in propagating UGW, it is observed that the longitudinal transducer directs most of its energy in setting up a standing wave along the *z*-axis at the excitation site, whereas the shear transducer more efficiently propagates guided wave modes given the same input amplitude. Therefore, shear type excitation is chosen as being the optimal configuration and will be used for the remainder of the work.

### 4.3. Operational Frequency Range

As previously explained (Section 2), operational frequency (or frequency range) is chosen for a high level of wave/damage interaction and effects visible from the top surface. This latter point is important since large modal conversions that are unobservable via the method of reception are ineffectual.

**Figure 7:**
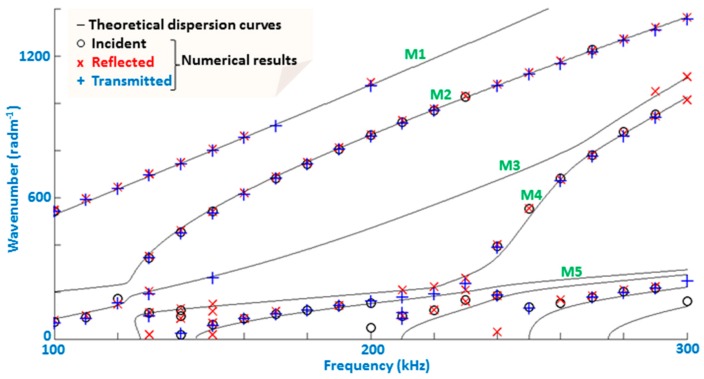
Locations of local maxima of the spatial Fourier transform of the incident (o), reflected (x) and transmitted fields (+) for a surface-originating crack type defect. Continuous black lines are the theoretical dispersion curves, plotted here to validate numerical results. The wavenumber is in absolute value to better compare which mode is which, even for reflected waves that in reality have a negative wavenumber [41].

Consider a surface originating crack, modeled as a straight-edged notch. Investigating all notch parameters is outside the scope of this paper, so instead, we pick a representative case of a crack having spread to 1/2 the thickness of the upper composite layer, i.e., 4 mm, located at *x* = 200 mm. The crack width is considered thin, so that the width of the notch in the FE model is one element. To optimize the input excitation frequency, we consider the incident, reflected and transmitted fields (I, R and T, respectively), as shown in Figure 3. A series of evenly spaced points are monitored on the top surface, via a spatial Fourier transform. Simulations were run from 100 to 300 kHz in increments of 10 kHz. The corresponding result is shown in Figure 7, which presents the distributions of modal amplitudes local maxima (above the threshold value of 1.5 times that of numeric noise) in the frequency-wavenumber diagram, for the three fields (I, R and T). This kind of presentation is suitable to check the presence of propagating modes [25]. The I and R field, although occupying the same physical space, are comprised of wave packets traveling in opposite directions. The incident waves are traveling to the right, whereas the reflected waves are obviously traveling to the left. Therefore, separation of the two fields is simply a matter of taking the correct portion of the frequency-wavenumber space spectrum.

**Figure 8:**
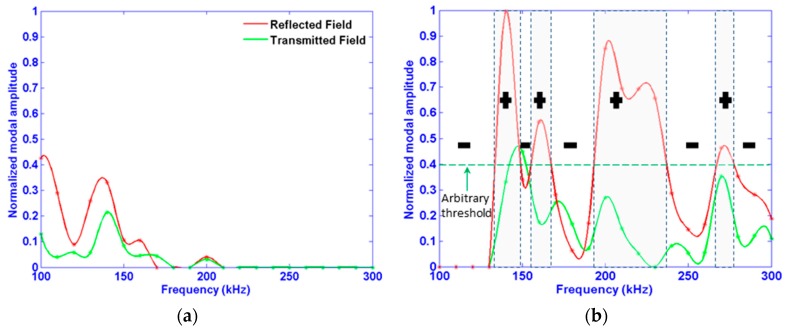
Mode 1 (**a**) and Mode 2 (**b**) amplitudes for the reflected (red) and transmitted fields (green) for a surface-originating crack type defect. All amplitudes are normalized by the maximum of the amplitude of Mode 2. The threshold is chosen arbitrarily to help identify the (−) and (+) frequency bands. Stars and continued lines are the collected values and the splined ones, respectively.

Figure 7 neatly displays which modes propagate in the I, R and T fields as a function of frequency. However, the optimal frequency for detection of a given mode is directly related to its amplitude, and so the amplitudes corresponding to the maxima of the two lowest order modes (Mode 1 and Mode 2) are shown in Figure 8. Accordingly, the said frequency range to most easily detect the given damage type can be determined. By examining this figure, the following information can be extracted:

- All modes do not have the same out-of-plane, surface amplitude (i.e., Mode 2 amplitude is more than three times that of Mode 1), either in transmitted or in reflected fields. In this case, Mode 2 is to be selected for experimental procedure.
- All modes depend on frequency, and do not behave similarly with regard to it. For example, the highest changes in modal amplitude occur in the reflective field around 100 kHz for Mode 1, whereas around 140 kHz, for Mode 2.
- An alternative of “like-pass” and “like-prohibited” frequency bands exist, in which mode amplitude is high and very low (quasi-null) respectively. They are indicated by (+) and (−) signs in the same figure, correspondingly. Note that this phenomenon has been reported by some researchers, such as Laguerre et al. for an embedded rod in a solid matrix, namely cement [42], Castaings et al. in the case of a pipeline containing a blockage [43], and Beard for a rock bolt [44]. This phenomenon is induced by constructive and destructive waves interferences combined with waves’ leakage in a surrounding medium (i.e., bilayer structure as it is the case in the current study). This phenomenon occurs in the reflected field as well as in the transmitted one. As a consequence, for the industrial procedure, the “like-pass” frequency bands are to be favored. In another words, “like-prohibited” frequency bands are to be avoided, to ensure efficient monitoring.
- By comparing modal amplitudes in the reflected and the transmitted fields, one can conclude that the receiving transducer should be placed in the reflective field if possible to detect the damage at hand. Pulse-echo measurement arrangement is better, in this case, than the pitch-catch one.

However, at this stage, the extent of the effect of phase of the incident wave remains unclear since the distance between the emitter and damage site has remained fixed. The next section will be devoted to investigating this topic, which could have a serious influence on the transducer placement and so implicitly, on monitoring cost and reliability.

### 4.4. Transducer Placement

The transducer placement is an important topic in the field of SHM. It has drastic repercussions on the SHM reliability as well as cost, if not well-investigated. In other words, if the number of transducers is under-estimated, it will be insufficient to cover the whole structure, and then some areas will never be monitored. If the number is over-estimated, the monitoring is reliable but too expensive. A trade-off should be found. Some studies were conducted to optimize the number and distribution of sensors [45]. Such an optimization method should take into account the material attenuation and damage characteristics. However, in the real-world, the location of damage is arbitrary and cannot be predicted accurately. What is the influence of the distance between the transducer and the damage on its detectability? Is the optimal frequency rage formerly assessed still robust, even for small distance variations (i.e., alteration of the phase of the incident wave)?

**Figure 9:**
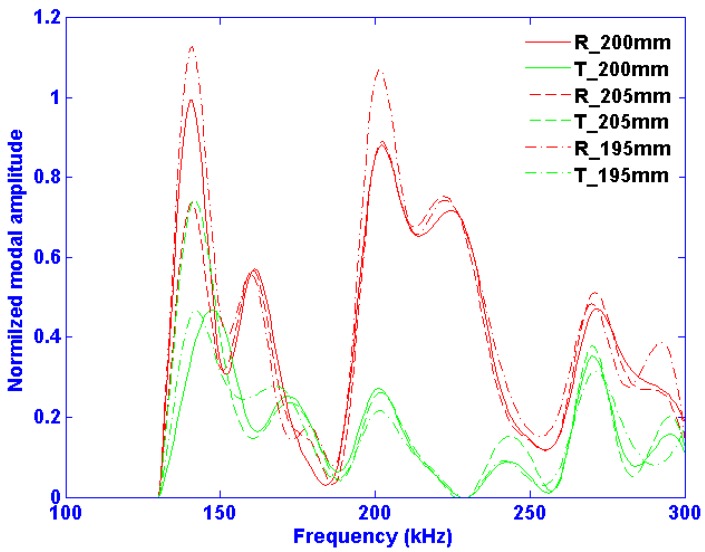
Same title as Figure 8 left but for different distances between the emitter and the crack (i.e., 195, 200 and 205 mm). R and T denote the reflected and the transmitted fields, respectively.

To answer this question, it is assumed that the sensors distribution is already optimized in the current study and only one sensor is used. The same FEM model is kept, but the distance from the sensor to the damage site is varied slightly (±2.5%). In a true experimental setup, obviously the damage location would be fixed and the transducers could move, thus changing the phase of the incoming wave. However, in a numerical environment the important component is that the phase changes at the moment of contact with the defect, and therefore can be realized by changing the location of the damage site. Many damage sites were simulated; Figure 9 shows the resulting Mode 2 amplitude for the cases where the notch is located at 195, 200 and 205 mm as an example case. These three locations correspond to the case where Mode 2 changes phase by roughly a quarter wavelengths at 140 kHz and half wavelength at 200 kHz. One can note qualitatively, that despite some minimal changes, it is clear that the damage detection method is rather robust to changes in the distance between the emitter and damage site.

The greatest amplitude change between the three methods is observable at the two frequencies that have the highest reflection coefficients, i.e., 140 and 200 kHz. At 200 kHz, when the defect site is at 195 and 205 mm (and the incident wave has evolved to being 360 degrees out-of-phase) the result is the same, which is to be expected. However, when the incident wave is 180 degrees out-of-phase, the overall amplitude drops by 16%.

At 140 kHz, all three cases are roughly 90 degrees (corresponding to a quarter wavelength) out of phase. The amplitude differences of 30% of these three cases are decidedly larger than at 200 kHz. However, despite these differences, one can note that the Mode 2 amplitudes as a function of frequency have similar trends, and this justly indicates a certain level of robustness in terms of changes in incident phase. Furthermore, the observable amplitude differences that do occur may have potential in precise damage localization with respect to transducer position, especially since the reflection and transmission fields’ changes are not directly proportional. For example, the reflection and transmission amplitudes are equal for the 205 mm case, whereas the reflection amplitude is more than double that of the transmission amplitude at 200 mm.

### 4.5. Transducer Shape, and Waves Propagation Analysis

Simulations are performed for two different kinds of excitation shape. The first one mimics a circumferential probe to excite the like-T(0,1) wave mode. This mode is privileged because it is non-dispersive in most cases [46]. A circumferential probe offers the possibility to generate this mode, and is easy to install by wrapping it around the COPV. This idea is inspired from existing commercial systems dedicated for pipelines, exploiting either piezoelectric [47] or magnetostrictive [48] effects, designed for long-term monitoring. A similar kind of probe but operating with PVDF effect has been investigated [49]. These systems could be adapted to monitor COPV structural integrity. In order to reach this aim, a comprehensive study is required.

The second type of excitation is local which simulates a piezo patch [50], a piezoelectric wafer active sensor [51], etc. Examples of application on other structure types, such as pipes and UAV wings can be found in the literature [52,53]. In the current case of application, COPV can be monitored through a sparse array of local transducers. In general, a local transducer permits monitoring by means of all types of possible propagating modes. These modes depend, as described earlier, on frequency range, excitation dimensions, and vibrations. Furthermore, this kind of transducer occupies less contact surface area with the COPV (by comparison with the global circumferential transducer), which causes lower risk of defect induced to the structure, or delamination from it.

**Figure 10:**
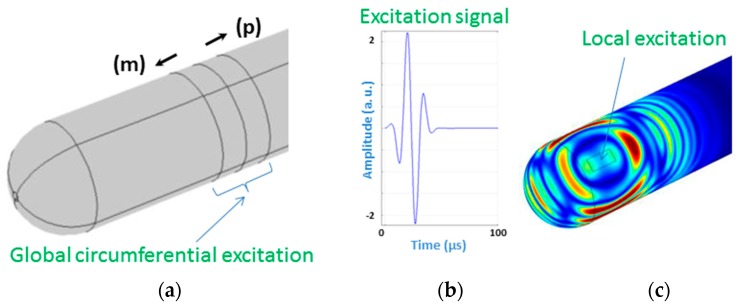
Transducer excitation: circumferential global excitation (**a**); the signal time of excitation (**b**) used for both excitations and local excitation (**c**). Note that the circumferential excitation consists of two parts where one is excited by the same signal (**b**) but shifted by a quarter of the main period.

A normal force load was applied in the area corresponding to the patch’s position. The excitation signal is a 3-cycle Gaussian pulse as shown in Figure 10.

#### 4.5.1. Circumferential Excitation

**Figure 11:**
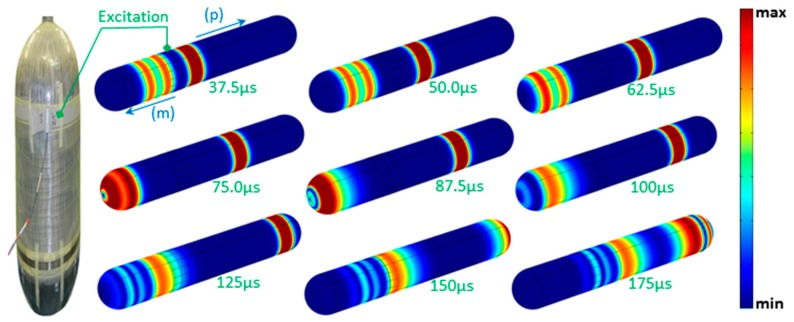
Photography of a vessel equipped with a circumferential actuator (**left**); and wave-field evolution for the like-T(0,1) mode, at different propagation time-steps (**right**). The signs (m) and (p) indicate negative and positive propagation directions, respectively.

According to the excitation principle, two force loads are added sequentially to two adjacent zones with equal axial length of λ/4, where λ is the wavelength. The two loads have the same time duration, but with a time shift of T_0_/4 one after the other, where T_0_ is the cycle decided by the exciting frequency. This configuration allows controlling the wave propagation direction; i.e., the wave propagating in the desired direction will be enhanced, while partly cancelled in the undesired direction, as it can be seen in Figure 11 indicated by the signs (m) and (p) [54]. This is ensured through exploiting the constructive and destructive phenomenon of waves. This technique is performed in order to localize in which side/direction, with respect to the transducer, a defect exists.

This figure (Figure 11) shows the evolution of the wave-field displacement during its propagation. Eight time-steps are selected and shown here; they cover the most representative steps of propagation, until the interaction with the end-caps and reflection back. The T(0,1)-like mode is generated, as can be seen in the same figure. The main signal characteristic is that two relative long-duration reflections occur due to continuous cross-section change, when the wave packet propagates in the semi-spherical shell. There are two reflections of this kind: one occurs when the wave propagates from the cylinder body to the geodesic end-cap for a continuous decrease of cross-section change; the other one occurs when the wave travels from the polar point of the edge to the cylinder body for a continuous increase of cross-section change. As a conclusion, analysis of waves generated and collected by the same kind of transducer (actuator/sensor) is straightforward, and this wave mode is useful in monitoring context as well as in testing.

**Figure 12:**
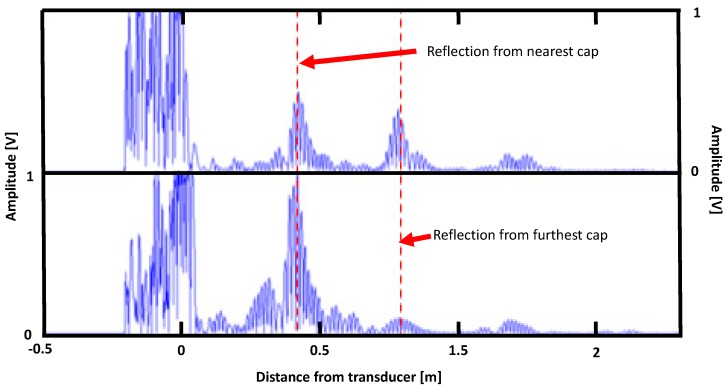
an experimental result corresponding to Figure 10, for a 64 kHz excitation on a real size vessel with the cylindrical part 43 cm long, with the transducer placed either on the cylinder, close to the cap (top of the figure); or on the cap itself (bottom of the figure).

For reasons of comparison for the main features occuring in a real experiment under similar conditions as in Figure 11, an experimental result is shown in Figure 12 for 64 kHz excitation on a real size vessel with the cilindrical part 43 cm long, with the transducer placed either on the cylinder, close to the cap (top of the figure), or on the cap itself (bottom of the figure). Reflected echoes are clearly visible caused by the caps in both locations and correspond to the features visible in Figure 11.

#### 4.5.2. Local Excitation

**Figure 13:**
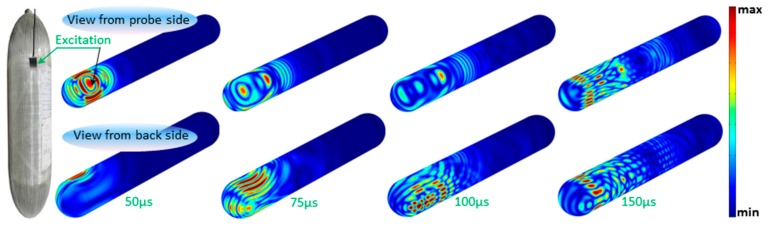
Wave field evolution in a vessel at different propagation time-steps.

The characteristics of the excitation used beforehand in the 2D frequency domain are kept in this study. Concerning its temporal shape, it is a 3-cycle Gaussian pulse. The wave-field displacement evolution, obtained after running computing, is exposed in Figure 13. Four time-steps are selected to show the waves’ behaviour in the top-side (view from probe side), and bottom-side (view from the back side). As can be seen in these results, the waves propagate in all directions and reverberantly go around the vessel resulting in interferences everywhere. The interference patterns correspond to the detected series of wave lobes.

**Figure 14:**
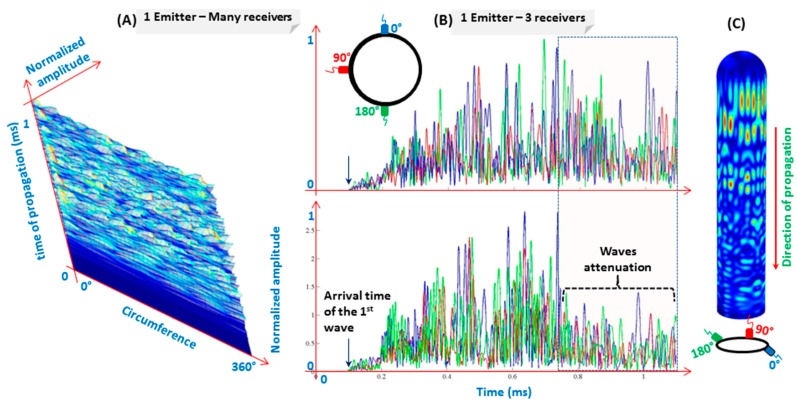
Tri-dimensional representation of signals amplitude versus angle of revolution and time of propagation (**A**); 3 waveforms collected at 0°, 90° and 180° (**B**) for two cases: with two end-caps; and with only one geodesic end-cap (**C**).

A ring of detection points (increment of 5°) is set around the cylinder circumference during the model construction to detect signals. Figure 14A shows a tri-dimensional representation of the signal amplitude versus time of propagation and the angle of revolution. As it can be expected based on Figure 13, the waveform varies with regard to the angle of revolution. For clearer representation and easier comparison between waveforms, Figure 14B (top) represents 3 signals taken at 0°, 90°, and 180°, which confirm the aforementioned phenomenon.

In order to better understand the waveforms (i.e., the origins of echoes), another simulation model was run with less interference, by eliminating the waves going back from the end-cap of the other side (i.e., the farthest from the excitation source). The model is so constructed with one geodesic end, while the other end becomes a cylindrical cross-section (see Figure 14C). This one is set as a low-reflection boundary. The use of these kinds of boundaries decreases drastically the amplitude of the reflected waves. A reliable selection of some parameters can allow simulating an infinite medium (see for example absorbing region in reference [55]). The simulation results show similar wave-field evolutions. To evaluate the influence of the end-cap on collected signals, Figure 14B (bottom) shows 3 signals, as in Figure 14B (top), to be effortlessly compared with. The main difference lies in the last 3rd part of the signals (see the area colored by light beige and with blue dashed boundary). This can be interpreted as: wave packets reflecting due to the geodesic end-cap are hardly detected. In the real-word, due to the large thickness of the end-cap (as it can be remarked in Figure 2), high waves attenuation should be expected, and so results are more similar to those of Figure 14B (bottom). These findings should be taken into account in experiments, for sensors and actuators distribution as well as analysis.

## 5. Conclusions

This paper dealt with hydrogen safety, through developing techniques and procedures to monitor and reliably test COPVs, envisioned for hydrogen storage. It expounded comprehensive investigations carried out in order to establish an efficient operational health monitoring procedure. Engineering analyses basing on numerical simulations in 2D and 3D domains have been achieved. The main conclusion is that UGW is a good candidate for testing and/or monitoring COPVs, and so contributing to the safety of hydrogen use. More technically, the foremost parameters to detect a specific defect type (surface-originating cracks which is the most dangerous kind of defects in COPVs) were identified and optimized. Specifically, it has been shown that:

- there is a significant and non-linear increase in the number of modes with the thickness and the number of layers;
- dispersion curves must be plotted on a case-by-case basis due to varying layer thickness ratios;
- a shear contact transducer was more efficient than longitudinal contact transducers at propagating UGW in the given thick COPV;
- modal amplitudes depend on frequency. Some frequency bands are more suitable than others. Some bands should be avoided;
- mode 2 appears to be the best mode for detecting the said defect type and that in pulse-echo measurement arrangement. This mode, although slightly dependent on the incident phase, was more or less robust to the distance between the emitter and damage site;
- concerning COPVs testing, further studies are needed for developing specific algorithms to help interpret signals, when operating local transducers;

These main findings will help to perform optimal experiments, which will be presented and debated in the second part of this paper.
